# Annual Research Review: Cash transfer programs and young people's mental health – a review of studies in the United States

**DOI:** 10.1111/jcpp.14101

**Published:** 2024-12-21

**Authors:** Sara R. Jaffee, George Lin, Matthew Z. Fowle, Vincent J. Reina

**Affiliations:** ^1^ Department of Psychology University of Pennsylvania Philadelphia PA USA; ^2^ Department of City and Regional Planning University of Pennsylvania Philadelphia PA USA

**Keywords:** Intervention, mental health, poverty, social policy

## Abstract

Worldwide, more than one in 10 children or adolescents is diagnosed with a mental disorder. Cash transfer programs, which aim to reduce poverty and improve life outcomes by providing direct cash assistance to families and incentivizing or enabling spending on education, health service use, dietary diversity and savings, have been shown to improve the mental health and well‐being of young people in low‐ and middle‐income countries. The goal of this review is to describe cash transfer programs in the United States, to describe potential mechanisms by which cash transfer programs could improve child and adolescent mental health and to summarize any evidence of the impact of cash transfer programs. We conclude that much of the evidence on the relationship between cash transfer programs and child and adolescent mental health in the United States is based on a relatively small set of studies. Although most of these studies find that cash transfer programs are associated with reductions in emotional or behavioural health problems, effect sizes are small. For potential mechanisms of cash transfer effects, the strongest evidence is that cash transfer programs increase child‐related expenditures and savings and increase time spent with children. Evidence is mixed on whether cash transfer programs improve maternal mental health, parental disciplinary practices or children's exposure to violence.

## Introduction

Worldwide, more than one in 10 children or adolescents is diagnosed with a mental disorder (Kieling et al., 2024; Polanczyk, Salum, Sugaya, Caye, & Rohde, 2015). In addition to evidence‐based treatments that target cognitions or emotion regulation, interventions that target social determinants of health may be effective at reducing rates of mental health problems (Cosgrove, Mills, Karter, Mehta, & Kalathil, 2020; Lund et al., 2018). For example, poverty has been widely studied as a contributor to poor mental health, with evidence from quasi‐experimental studies that it is a cause of depression, anxiety and posttraumatic stress disorder (Marchi et al., 2024) and that transitions out of poverty are associated with increases in well‐being and decreases in mental health problems (Thomson et al., 2022). Our goal is to review and synthesize the evidence that poverty reduction programs – specifically, cash transfer programs – reduce mental health problems in young people (whom we define as persons under 19 years) in the United States. We first review the evidence from quantitative meta‐analyses of poverty reduction programs from mostly low‐ and middle‐income countries. We then describe cash transfer programs in the United States and the evidence that these programs improve mental health for children and adolescents. We also review the evidence for potential mechanisms by which cash transfer programs improve young people's mental health. Finally, we summarize lessons learned from cash transfer programs in low‐ and middle‐income countries as well as the United States and describe future directions for research.

Although most cash transfer studies have been conducted in low‐ and middle‐income countries, such programs are growing in popularity in high‐income countries like the United States. There is a need to understand whether and how they affect young people's mental health. As noted by Shah and Gennetian (2023), findings from low‐ and middle‐income countries may not generalize to the US context for at least two reasons. First, cash transfer programs raise a family's income substantially in low‐ and middle‐income countries (by as much as 200%; West, Castro, & Doraiswamy, 2023), but in the United States, the increase in family income associated with cash transfers is typically more modest because earnings are much higher, even amongst poor families. Second, because poor people in the United States have access to a patchwork of services, there is a need to understand the impact of cash payments in the context of a lack of access to a comprehensive social safety net. These findings call for a focus on the impact of cash transfer programs in the United States specifically.

## Cash transfer programs in low‐ and middle‐income countries

Globally, about 1.5 billion people live in poverty, with 61% of those people under the age of 24 years (Robles Aguilar & Sumner, 2020). In 2019, 23% of the global population lived on less than US$3.65 per day and 47% lived on less than US$6.85 (Baahr et al., 2023). In response, cash transfer programs began in the late 1990s as a strategy to reduce poverty in low‐ and middle‐income countries and thereby improve life outcomes (Bastagli et al., 2016; The World Bank, 2018).

In general, cash transfer programs aim to reduce poverty by providing direct cash assistance to families and to improve life outcomes by incentivizing or enabling spending on education, health service use, dietary diversity and savings. Cash transfer programs typically take one of two forms. In conditional cash transfer (CCT) programs, receipt of cash payments is conditional on households complying with program requirements (e.g., that children attend school or receive preventative healthcare). In unconditional cash transfer (UCT) programs, receipt of cash payments is unconditional, meaning a household is not required to comply with a program requirement to receive partial or full cash payments. Cash transfer programs may involve either one‐time, lump sum payments (e.g., the Earned Income Tax Credit, which is paid in the form of a tax refund) or recurring payments (e.g., cash transfer programs that pay participants monthly over some period of time).

Today, cash transfer programs are widely available in Africa, Latin America and Asia (Bastagli et al., 2016), where their impact has been demonstrated across measures of monetary poverty, education, health, empowerment (Bastagli et al., 2019) and adult mental health (Lund et al., 2011; McGuire, Kaiser, & Bach‐Mortensen, 2022; Ridley, Rao, Schilbach, & Patel, 2020; Wollburg, Steinert, Reeves, & Nye, 2023). Two meta‐analytic reviews of experimental and quasi‐experimental studies of cash transfer programs have focused on symptoms of depression and anxiety in children and adolescents. One showed significant effects in reducing symptoms of internalizing problems in children and adolescents under the age of 20 years (Zaneva, Guzman‐Holst, Reeves, & Bowes, 2022). The other showed small and nonsignificant effects in reducing symptoms of depression in young people under the age of 25 years, with the studies of depression symptoms showing significant variability in effect size (Zimmerman et al., 2021).

## Cash transfer programs in the United States: Effects on child and adolescent mental health

In the United States, support for low‐income families is primarily in the form of in‐kind transfers such as the Supplemental Nutrition Assistance Program (SNAP), Medicaid and housing assistance (e.g., the Housing Choice Voucher Program; Pilkauskas, Jacob, Rhodes, Richard, & Shaefer, 2023). In‐kind transfers have demonstrable benefits for families (Ellen, 2020; McKernan, Ratcliffe, & Braga, 2021; Schwartz, Horn, Ellen, & Cordes, 2020; Shaefer & Gutierrez, 2013) and there is growing interest in the United States in combining these forms of assistance with conditional and unconditional cash payments (Gennetian, Shafir, Aber, & De Hoop, 2021; National Academies of Science, Engineering, and Medicine, 2019).

Cash transfer programs typically take one of three forms in the United States: (1) tax credits for lower‐income working families, including the Earned Income Tax Credit and the Child Tax Credit; (2) payments to eligible households from industry‐related cash distributions, such as payments to Native American households from casino profits or to Alaska state residents from the trans‐Alaska oil pipeline and (3) and direct cash payments to low‐income and other vulnerable groups through guaranteed income programs. We describe these in turn and review the evidence from quasi‐experimental and experimental studies that these programs are associated with reductions in children's emotional and behavioural health problems.

Our literature review was conducted from October 2023 through January 2024. First, we identified three recent reviews of tax credits, other cash transfers and child and adult outcomes in high‐income countries (Boccia et al., 2023; Shah & Gennetian, 2023; Simpson, Albani, Bell, Bambra, & Brown, 2021). From those reviews, we then identified all papers focused on tax credits or other cash transfers (e.g., industry‐related cash distributions) and mental health in children and adolescents (under age 19 years); we read those for additional relevant citations. Our definition of mental health included any DSM‐5 (Diagnostic and Statistical Manual‐5) disorders measured at the symptom or the diagnosis level (American Psychiatric Association, 2022), broadband measures of internalizing and externalizing problems (reflecting depressed, anxious and withdrawn behaviour or aggressive and rule‐breaking behaviour) and single items reflecting emotional or behavioural health problems (e.g., physical fighting).

Next, we used Web of Science and Google Scholar to locate papers that cited any of those identified in the first two stages and we continued these forward searches on newly identified papers until no new papers were identified. Finally, we searched Web of Science, Google Scholar, National Bureau of Economic Research and the Social Science Research Network using the keyword ‘guaranteed income’, narrowed the selection to studies of guaranteed income that focused on mental health in children and adolescents, and then used Web of Science and Google Scholar to identify any additional papers that cited the ones located. We focused on cash transfer programs implemented in the United States, although we highlighted one study from Canada (Milligan & Stabile, 2011) and another from the United Kingdom (Beatty, Blow, Crossley, & O'Dea, 2014) for comparison.

### Findings from studies of tax credits

As Shah and Gennetian (2023) describe in their scoping review of cash transfer programs in the United States, tax credits, distributed through the tax system, are the largest source of cash aid to families in the United States, with the tax system providing an efficient vehicle for delivery of payments and for verification of eligibility. In the United States, families with children potentially benefit from two tax credits: the Earned Income Tax Credit (EITC) and the Child Tax Credit (CTC).

#### Earned Income Tax Credit

The EITC is available to low‐ and moderate‐income workers and is designed to encourage work by offsetting federal payroll and income taxes as wages increase, although the EITC ‘phases out’ once incomes reach a certain level (Center on Budget and Policy Priorities, 2023). If the value of the credit exceeds what the recipient owes in federal taxes, the difference is delivered to the recipient as a tax refund. The amount of the EITC depends on an individual's income, marital status and the number of children, with more generous credits for working families with children. In 2018, the EITC lifted 5.6 million people out of poverty based on the Supplemental Poverty Measure, which takes into account not only income from all sources but also refundable tax credits and in‐kind benefits as well as taxes and nondiscretionary expenses (Center on Budget and Policy Priorities, 2023).

Research on the impact of the EITC on child and adolescent mental health has typically used a range of quasi‐experimental approaches. One is the difference‐in‐differences method, which compares groups before and after one of the groups (but not the other) would presumably have benefited from a change in EITC policy. Evidence for a significant effect of the change in policy would be found if children whose families presumably benefited from the policy change (e.g., by receiving higher credits) showed a reduction in mental health problems from before to after the policy change and relative to those children whose families did not benefit from the policy change. As an example, the 1993 expansion of the EITC resulted in appreciably higher credits for families with two or more children (vs. one child), suggesting that children with one or more siblings should have shown greater declines in mental health problems from before to after the expansion than children without siblings in the same time period.

An analysis of the 1993 expansion of the EITC, however, using data from the National Longitudinal Survey of Youth 1979 Child and Young Adult (NLSY79CYA) sample from the years 1993 to 1998 found that it was not associated with declines in emotional and behavioural problems (as measured with the Behaviour Problems Index; Peterson & Zill, 1986) from before compared to after the expansion (Averett & Wang, 2018). Batra and Hamad (2021) used data from the National Health Interview Survey (NHIS) to explore the short‐term effects of EITC refund receipt on children's emotional and behavioural health, as measured by an adaptation of the Child Behaviour Checklist (Achenbach & Rescorla, 2000) for 2–3‐year‐olds and the Strengths and Difficulties Questionnaire (Goodman, 1997) for 4–17‐year‐olds (*n* = 172,281). This study used the difference‐in‐differences method to exploit naturally occurring variation in the month in which families were interviewed for the NHIS relative to receipt of the EITC refund, such that some parents were interviewed about their child's health and behaviour shortly after receiving the refund (e.g., between February and April) and other parents were interviewed about their child's health and behaviour at other times of the year (e.g., between May and June). Children whose families were interviewed shortly after receiving the refund did not have significantly lower levels of emotional and behavioural problems compared with children whose families were interviewed at other times of the year and, as expected, children's emotional and behavioural problems did not vary as a function of interview timing for children whose families were not eligible for the EITC (Batra & Hamad, 2021).

In contrast, in a sample of 4–14‐year‐olds from the NLSY79CYA sample, changes in the value of EITC payments between the years 1986 and 2000, were associated with improvements in children's emotional and behavioural problems (as measured with the Behaviour Problems Index; Peterson & Zill, 1986). Relatively larger EITC payments in a given year were associated with lower child emotional and behavioural problems at 2 years (but not 4 years) follow‐up (Hamad & Rehkopf, 2016). Finally, a different approach to estimating the impact of the EITC has been to exploit state‐level variation in the structure and generosity of EITC benefits. Using cross‐sectional data from 9th through 12th graders interviewed for the Youth Risk Behaviour Surveillance System between 2005 and 2019, Dalve et al. (2022) found that youth violence for boys but not girls was lower in states with more generous EITC credits. However, the study's state‐level difference‐in‐differences analysis is unable to offer insight on the role of EITC payment amount or the mechanisms that might produce these effects and does not facilitate within‐person comparisons.

In summary, findings relating the EITC to children's emotional and behavioural health problems are mixed, even in studies that used the same set of data over approximately the same time frame (Averett & Wang, 2018; Hamad & Rehkopf, 2016). It is possible that different analytic approaches generated different findings. Hamad and Rehkopf (2016) used variation in the size of the EITC credit (ranging from $0 for families who were ineligible to the maximum EITC credit value for eligible families) over time as an instrumental variable. In contrast, Averett and Wang (2018) used a difference‐in‐differences approach based on the large increase in EITC benefits for families with at least two children compared to those with one child. They estimated the impact of EITC receipt (yes or no) prior to versus after the 1993 expansion rather than over several years. Both studies estimate EITC eligibility: Averett and Wang (2018) use years of mothers' education, whereas Hamad and Rehkopf (2016) use household income and demographic characteristics. These analytic strategies introduce estimation bias, in addition to both studies' assumptions of full program take‐up. It may also be the case that benefits of credit receipt for child and adolescent mental health take time to accrue. For example, data from the cross‐sectional NHIS series failed to show short‐term impacts of EITC payments on children's emotional and behavioural health (Batra & Hamad, 2021), but data from the longitudinal NLSY79CYA showed effects of EITC credit size on children's emotional and behavioural problems 2 years later (Hamad & Rehkopf, 2016).

#### Child Tax Credit

Like the EITC, the CTC is available to low‐ and moderate‐income households, although only households with children under the age of 17 years are eligible. The CTC is designed to offset child‐care costs (although there are no restrictions on expenditures) and, like the EITC, phases out when adjusted gross income reaches a certain level. The CTC has been expanded on multiple occasions, originally in 2001 when it was made partially refundable for families earning at least $10,000 and again in 2009 when it was made partially refundable for families earning at least $3,000. Like research on the EITC, research on the CTC typically uses difference‐in‐differences methods to test whether changes to CTC policy, either resulting in more families becoming eligible for credits or higher credits for families, are associated with changes in children's mental health. For example, in an analysis of data from the National Longitudinal Survey of Youth 1979 (NLSY79) and the NLSY79CYA, changes to the CTC in 2009 (making it partially refundable for families earning as little as $3,000) were associated with reductions in children's emotional and behavioural problems, as measured by the Behaviour Problems Index (Rostad, Klevens, Ports, & Ford, 2020). Reductions in behavioural problems were significantly larger for boys, especially in early childhood. In contrast, a quasi‐experimental study of the Canada Child Tax Benefit (less generous than the US CTC but without an income floor for eligibility) found children in families receiving larger transfers had significant improvements in mental health, which were largest for girls (Milligan & Stabile, 2011).

### Findings from studies of industry‐related cash distributions

In a few settings, a local economic development effort has tied business profits to a cash benefit distributed directly to local community members. In one case, tribal members of the Eastern Band of Cherokee Indians used the 1996 opening of a gambling casino on their reservation to provide each child and adult in the community approximately $6,000 annually from the casino profits (Costello, Compton, Keeler, & Angold, 2003). Native American children who were 9, 11 or 13 years old in 1993 and whose families were lifted out of poverty by the opening of the casino showed a significant decrease in mean levels of psychiatric symptoms (as measured by the Child and Adolescent Psychiatric Assessment; Angold & Costello, 2000) from 4 years before the casino opened in 1996 to the 4 years afterwards. In contrast, mean levels of psychiatric symptoms remained high for Native American children whose families were not lifted out of poverty and low for those whose families were never in poverty (Costello et al., 2003). In young adulthood, receipt of casino cash payments was associated with a reduced risk of any psychiatric disorder (particularly, alcohol and cannabis use disorders) with the strongest effects observed amongst the youngest cohort who had the longest exposure to increased income (Costello, Erkanli, Copeland, & Angold, 2010). Casino cash payments were also associated with a reduced risk of engaging in minor criminal offenses and drug dealing by young adulthood when respondents were 19 or 21 years old (Akee, Copeland, Keeler, Angold, & Costello, 2010). The impact of cash payments on these outcomes was greatest for young people whose families were the poorest before the casinos opened. Follow‐up studies report that reductions in anxiety, depressive and cannabis‐related symptoms and risky behaviours persist through age 30 (Akee, Copeland, & Simeonova, 2024; Copeland et al., 2022).

### Findings from unconditional and conditional cash transfers (nontax credit)

The kinds of conditional and unconditional cash transfer programs that target poor and vulnerable populations in low‐ and middle‐income countries have historically been rare in the United States (Handa et al., 2018). In recent years that has changed, with cash transfer pilots and programs being developed across the country. We describe one conditional and several unconditional cash transfer programs below.

Opportunity NYC/Family Rewards was the first conditional cash transfer program in a high‐income country (Riccio et al., 2013). Launched in 2007, it was designed to incentivize parents' and children's participation in healthcare, education and employment and thereby build human capital (Riccio et al., 2013). Families who were eligible for Family Rewards lived in selected community districts, had incomes at or below 130% of the federal poverty threshold and had at least one child in fourth, seventh or ninth grade in 2007 (although all children who were school‐age or younger were included in the study once the family volunteered to participate). Almost all eligible families were Hispanic or Black. Families were randomly selected for the Family Rewards Program from those who applied. Evaluation of Family Rewards involved 4,800 families with 11,000 children, half of whom were assigned to the treatment group and half to the control group. Over the 3 years of the pilot, families received $8,700, on average. Like other cash transfer programs, Family Rewards reduced material hardship whilst families participated in the study. The program increased monthly income by about 22% relative to the control group and reduced the percentage of families who were living below the federal poverty threshold, although these impacts did not persist once the program ended (Riccio et al., 2013).

Of the 1,028 families with a ninth grader at random assignment, a subsample of 716 was selected for interviews about adolescent emotional and behavioural health; 511 adolescent–parent dyads completed these interviews in the second year of the 3‐year Family Rewards program. Family Rewards had no impact on self‐reported depression or anxiety symptoms as measured by the Behaviour Assessment System for Children (Reynolds & Kamphaus, 1992), but adolescents in the treatment group engaged in significantly less aggressive behaviour, alcohol and marijuana use, and had fewer friends who drank alcohol or used marijuana compared with adolescents in the control group (Morris, Aber, Wolf, & Berg, 2017).

During the COVID‐19 pandemic, various organizations (including the federal government through a series of economic impact payments) made unconditional, lump‐sum payments to households. In May 2020, the charitable organization GiveDirectly provided a one‐time, lump sum payment of $1,000 to 7,915 families with children who were receiving or had recently received SNAP benefits and had consented to participate in a research study about the impact of the COVID‐19 pandemic on their family (Pilkauskas et al., 2023). An additional 5,777 families with children were randomly assigned to the control group, which did not receive the $1,000 payment. Families completed surveys at baseline and at 1 and 3 months postbaseline. Although the $1,000 payment reduced material hardship at 1 month amongst the lowest‐income families, the payment was not associated with reductions in child problem behaviours, as measured by the Child Behaviour Checklist (Achenbach & Rescorla, 2000; Pilkauskas et al., 2023).

Guaranteed income programs provide unconditional, monthly payments of a set amount to eligible households. To the best of our knowledge, the only guaranteed income pilot designed as a randomized controlled trial that includes children and published data as of January 2024 on child outcomes is Baby's First Years (BFY), which includes 1,000 parents who were recruited from hospitals shortly after giving birth between July 2018 and June 2019 in New Orleans, New York City, the Twin Cities and the greater Omaha area (Noble et al., 2021). To date, findings related to children's emotional and behavioural health are not available in BFY. Researchers have however published findings on early life biobehavioural outcomes that are predictive of child and adolescent emotional and behavioural health, such as sleep and EEG recordings. On the one hand, the size of the monthly cash payment ($333 vs. $20) was not associated with parent reports of children's sleep, health or healthcare utilization at 1, 2 or 3 years of age (Sperber et al., 2023). On the other hand, infants whose parents received the higher cash gift showed more EEG activity in higher‐frequency bands (associated with the development of cognitive skills) than infants whose parents received the lower cash gift (Troller‐Renfree et al., 2022).

### Summary of cash transfer programs in the United States and child and adolescent mental health

Strikingly few quasi‐experimental or experimental studies of various forms of cash transfer programs in the United States focus on child and adolescent mental health. The studies we reviewed (Table 1) provide mixed evidence that cash transfer programs reduce children's and adolescents' emotional and behavioural problems. There is some evidence that expansions of the EITC and CTC were associated with small reductions in these problems and evidence of larger effects on adolescent mental health from an industry‐related cash distribution study. Amongst adolescents, these effects were more pronounced for substance use and antisocial behaviour than for symptoms of depression and anxiety. Evaluations of programs offering one‐time, lump‐sum payments during the COVID‐19 pandemic produced null results.

**Table 1:** Summary of findings from cash transfer studies on child and adolescent mental health in the United States

Study	Type of cash transfer	Sample	Mental health measure	Results
Averett and Wang (2018)	EITC	National Longitudinal Survey of Youth 1979 Child and Young Adult sample from 1993 to 1998 *n* = 7,328 Age: *M* = 7.53, *SD* = 3.68	BPI	*Null*: The 1993 expansion was not associated with declines in children's emotional and behavioural health problems
Batra and Hamad (2021)	EITC	National Health Interview Survey from 1998 to 2016 *n* = 172,281 Age: 2–17‐year‐olds	CBCL (adapted) and SDQ	*Null*: Children whose families were interviewed shortly after receiving the refund did not have significantly lower levels of emotional and behavioural problems compared with children whose families were interviewed at other times of the year
Hamad and Rehkopf (2016)	EITC	National Longitudinal Survey of Youth 1979 Child and Young Adult sample *n* = 8,186 Age: 4–14‐year‐olds	BPI	*Positive*: Relatively larger EITC payments in a given year were associated with significantly lower child emotional and behavioural problems at 2 years (but not 4 years) follow‐up
Dalve et al. (2022)	EITC	Youth Risk Behaviour Surveillance System between 2005 and 2019 *n* = 26–46 states Age: 9–12th graders	Youth violence	*Positive*: Youth violence was significantly lower in states with more generous EITC credits
Rostad et al. (2020)	CTC	National Longitudinal Survey of Youth 1979 (NLSY79) and the NLSY79 Child and Young Adult Survey *n* = 11,521 Age: 4–17 years	BPI	*Positive*: Changes to the CTC in 2009 (making it partially refundable for families earning as little as $3,000) were associated with reductions in children's emotional and behavioural problems
Costello et al. (2003)	Casino profits for tribal members	Great Smoky Mountain Study *n* = 1,420 Age: 9, 11 or 13 years old in 1993; comparison of psychiatric symptoms from before the casino opened (1993–1996) to after (1997–2000)	CAPA	*Positive*: Native American adolescents whose families were lifted out of poverty by the opening of the casino showed a significant decrease in mean levels of psychiatric symptoms. In contrast, mean levels of psychiatric symptoms remained high for Native American children whose families were not lifted out of poverty and low for those whose families were never in poverty
Costello et al. (2010)	Casino profits for tribal members	Great Smoky Mountain Study *n* = 1,420 Age: 12, 14 or 16 years old in 1996; psychiatric assessment at 19 or 21 years	CAPA and YAPA	*Positive*: Receipt of profits was associated with a reduced risk of any psychiatric disorder (particularly, alcohol and cannabis use disorders) with the strongest effects observed amongst the youngest cohort who had the longest exposure to increased income
Akee et al. (2010)	Casino profits for tribal members	Great Smoky Mountain Study *n* = 1,420 Age: 9, 11 or 13 years old in 1993; crime measured from age 16 to 21 years	Crime, measured by North Carolina Administrative Office of the Courts	*Positive*: Casino profits were associated with reduced risk of engaging in minor criminal offenses and drug dealing
Morris et al. (2017)	CCT	Family Rewards; 262 program group 249 control group; Age: *M* = 16.6 years, *SD* = 0.83)	BASC Items measuring minor delinquency, aggression, and substance use	*Null*: No impact on self‐reported depression or anxiety symptoms *Positive*: Adolescents in the treatment group engaged in significantly less aggressive behaviour, alcohol and marijuana use, and had fewer friends who drank alcohol or used marijuana compared with adolescents in the control group
Pilkauskas et al. (2023)	UCT	7,915 cash group 5,777 control group	Modified CBCL	*Null*: Although the $1,000 payment reduced material hardship at 1 month amongst the lowest‐income families, the payment was not associated with reductions in child problem behaviours

BASC, Behavioural Assessment System for Children; BPI, Behaviour Problems Index; CAPA, Child and Adolescent Psychiatric Assessment; CBCL, Child Behaviour Checklist; CCT, Conditional Cash Transfer; EITC, Earned Income Tax Credit; SDQ, Strengths and Difficulties Questionnaire; UCT, Unconditional Cash Transfer; YAPA, Young Adult Psychiatric Assessment.

## Mechanisms of cash transfer programs

As noted earlier, mental health and psychological well‐being are typically secondary outcomes in existing cash transfer programs. As such, these programs are rarely designed to identify mechanisms by which these outcomes improve. Nevertheless, there are existing theoretical frameworks for thinking about links between poverty alleviation and young people's emotional and behavioural health and, as reviewed by Evans‐Lacko et al. (2023), qualitative data from existing studies suggest additional mechanisms. In this section, we describe potential mechanisms and review the evidence that cash transfers are effective in changing these factors. These mechanisms are not mutually exclusive and may reflect more proximal versus distal explanations for program effects.

### Increased income leads to greater cash and time investments in children

One prominent explanation for why increases in income are associated with improvements in children's outcomes is the *family investment theory*, which hypothesizes that increases in income allow parents to invest more in material resources for children and to spend more time with children (Becker & Thomes, 1986; Haveman & Wolfe, 1995). There is consistent evidence from the programs reviewed in the previous section that cash transfers increase spending on child‐related goods and services, with some programs designed to promote such spending (e.g., Family Rewards and Baby's First Years). For example, in the Baby's First Years Study, mothers in the higher cash group spent 20% more on child‐related expenditures in the month prior to the survey when their infant was 1 year old than mothers in the lower‐cash group – a difference that was statistically significant (Gennetian et al., 2022). Other analyses of spending data show that child education‐related expenditures increase (particularly for low‐ and middle‐income parents) in the months immediately surrounding Alaska Permanent Fund dividend payments which, since 1982, have been made annually to Alaska residents to return earnings from the trans‐Alaska oil pipeline (Amorim, 2022). Two of three studies that looked at spending in the context of the expanded Child Tax Credit reported increases in child‐related expenditures (Schild et al., 2023), including spending on groceries, education and healthcare (Lourie, Shanthikumar, Shevlin, & Zhu, 2022). In contrast, a study of approximately 7,000 New York City residents enriched for families with young children did not find that child‐related expenditures increased in the months when CTC payments were issued compared with the previous months (Collyer et al., 2022).

In addition to enabling families to invest more in material resources, cash payments may enable families to spend more time together. Particularly in the United States where access to benefits is conditional on employment status, many parents with limited education and training often work multiple low‐paying jobs to make ends meet, with over 8 million Americans working more than one job (US Bureau of Labor Statistics, 2024). Cash transfer programs could increase the amount of time available for parents to spend with children by enabling them to work fewer jobs. In the Baby's First Years Study, mothers in the higher cash group reported spending significantly more time engaging in child‐enriching activities (like reading books or telling stories to their infants) compared with mothers in the lower cash group, although effect sizes were small in magnitude (Gennetian et al., 2022). In interviews with parents in the Stockton Economic Empowerment Demonstration, which enrolled 125 low‐income households in Stockton, California in 2019 and provided an unconditional, monthly payment of $500 for 24 months, those who received monthly payments noted that they had more time to spend with their children doing every day and pleasurable activities, like helping with homework (West, Castro Baker, Samra, & Coltrera, 2021). Adolescents in the randomized Family Rewards treatment group were more likely than adolescents in the control group to spend the majority of their time with family members (or a combination of family members and peers) than to spend the majority of their time with peers (Morris et al., 2017).

Other studies, however, have not identified an effect of cash payments on time spent with children. Using seasonal variation in EITC payment receipt (i.e., comparing households in months when the majority of refunds are disbursed vs. months when federal outlays are lower), researchers detected few short‐term effects on time spent with children or on time spent in specific activities with children (Morrissey, 2023). Data from the 2003–2018 American Time Use Surveys found that variation in EITC benefits across states and across family size was unrelated to time spent on activities like homework, reading or engaging in educational activities (Bastian & Lochner, 2020).

In summary, there is consistent evidence from conditional and unconditional cash transfer programs that increased income allows parents to increase child‐related expenditures, including spending on education‐related activities, college savings, toys, clothing groceries and healthcare. These goods and services are likely to directly affect young people's emotional and behavioural health (e.g., by reducing food insecurity or enabling parents to pay for needed mental health services; Hatem et al., 2020; Shankar, Chung, & Frank, 2017). They are also likely to be indirectly associated with young people's emotional and behavioural health via effects on school‐related outcomes (Yeung, Linver, & Brooks–Gunn, 2002). Such spending may promote school readiness (e.g., through the provision of high‐quality child care), provide academic enrichment or remedial assistance (e.g., tutoring and extracurriculars), enable children to maintain attention (e.g., because they are not distracted by hunger or other poverty‐related concerns) and reduce absenteeism (e.g., through medications and preventative medical services that keep children physically healthy). Children who are able to achieve academically (because of these factors) are at lower risk of emotional and behavioural health problems, although the evidence is as strong or stronger for bidirectional effects (van der Ende, Verhulst, & Tiemeier, 2016).

Moreover, this literature is largely consistent in showing that conditional and unconditional cash transfer programs (including tax credits) increase the amount of time parents spend with children, engaging in cognitively stimulating activities like reading books and on every day, pleasurable activities that are hypothesized to promote parent–child bonding. Time spent together in these ways may mitigate risk for emotional and behavioural problems by promoting children's internalization of behavioural norms (Kochanska, 2002), increasing opportunities for caregivers to provide emotional support and build children's emotional understanding (Brownell, Svetlova, Anderson, Nichols, & Drummond, 2013), reducing opportunities for older youth to engage in substance use or risky behaviour with peers (Aber, Morris, Wolf, & Berg, 2016) or by increasing parents' ability to monitor their children (although evidence for this mechanism is lacking; Morris et al., 2017).

Finally, cash transfers may also mean adolescents do not need to contribute, or can contribute less, to household income, thus freeing up their time. Adolescents who have more intense part‐time work schedules during the school year spend less time engaged in school activities and are more likely to engage in problematic substance use (Monahan, Lee, & Steinberg, 2011; Safron, Schulenberg, & Bachman, 2001). Data are needed to formally test the hypothesis that increased spending on child‐related expenditures and increased time spent with children (or increased time for children to spend with family members or on school work) are mechanisms by which cash transfer programs promote positive emotional and behavioural health and to test hypotheses about the specific mechanisms of action.

### Increased income relieves financial stress and improves mental health of caregivers, parent–child interactions and intimate partner relations

A second prominent explanation for why increases in income are associated with improvements in children's outcomes is *family strain theory*, which hypothesizes that increases in income reduce stress related to housing and food insecurity and financial precarity, thereby improving caregiver well‐being and reducing unhealthy coping strategies, resulting in more positive parent–child interactions (Conger & Elder, 1994). The family strain theory also predicts that reductions in financial stress will improve intimate partner relations, which may result in more positive parent–child interactions that are associated with lower levels of emotional and behavioural problems (Conger & Elder, 1994). Some support for this model comes from mediation analyses of cash transfer programs in low‐ and middle‐income countries showing, for example, that cash transfers reduce maternal depression by alleviating perceived stress (Ozer, Fernald, Weber, Flynn, & VanderWeele, 2011), reduce health risk behaviours (Ohrnberger, Anselmi, Fichera, & Sutton, 2020) and improve youth mental health partly via increases in maternal well‐being (Angeles et al., 2019). In this section, we review the evidence that cash transfer programs in the United States (1) improve caregiver well‐being, (2) improve intimate partner relations and (3) improve parent–child interactions.

#### Parent well‐being

There is a growing body of evidence that cash transfer programs in low‐ and middle‐income as well as higher‐income countries improve maternal well‐being. A meta‐analysis of 45 experimental and quasi‐experimental studies conducted in Africa, Latin America and Asia, and involving 116,999 individuals, found that there was a small, but statistically significant association between cash transfers and both subjective well‐being (Cohen's *d* = .13) and mental health (Cohen's *d* = .07), with the effect increasing the greater the size of the transfer relative to family income (McGuire et al., 2022).

In high‐income countries, it is less clear whether cash transfers lead to reductions in parents' emotional and behavioural health problems. Results from evaluations of one‐time, lump sum payments made during the COVID‐19 pandemic show no effects on symptoms of depression and anxiety (Jacob, Pilkauskas, Rhodes, Richard, & Shaefer, 2022; Pilkauskas et al., 2023) or participants' reports of psychological well‐being (reflecting agency, subjective well‐being and symptoms of depression; Jaroszewicz, Jachimowicz, Hauser, & Jamison, 2022). In the Baby's First Years Study, mothers in the higher and lower cash groups did not differ when their infants were 1 year old with respect to self‐reported agency and hope (Gennetian et al., 2022), psychological distress (Magnuson et al., 2022) or alcohol or opioid use (which was low for both groups) (Yoo et al., 2022).

In contrast, expansion of the CTC during the COVID‐19 pandemic was associated with reductions in symptoms of depression and anxiety amongst low‐income parents, including for 800,000 Americans earning under $35,000 (Batra, Jackson, & Hamad, 2023) and over 15,000 Americans earning under $10,000 (Kovski, Pilkauskas, Michelmore, & Shaefer, 2023). The CTC expansion was also associated with reductions in mild symptoms of depression and anxiety in a nationally representative sample of over 160,000 American adults with children (Cha, Lee, & Tao, 2023). However, two studies found the 2021 CTC was not associated with decreases in adults' psychological distress as measured in a sample of over 7,000 New Yorkers (Collyer et al., 2022) or in a nationally representative sample of over 7,000 Americans (Glasner, Jiménez‐Solomon, Collyer, Garfinkel, & Wimer, 2022). In Family Rewards, parents in the treatment group reported significantly higher levels of hope (reflecting agency and goal‐directedness) than control group families shortly after the program ended, although the effect size was small and treatment effects were not observed on a measure of psychological distress that captured symptoms of depression and anxiety (Courtin et al., 2018; Riccio et al., 2013). In the Stockton Economic Empowerment Demonstration, adults in the treatment group reported significantly lower levels of depression and anxiety than adults in the control group in the first year of the study (from December 2018 until February 2020), but the groups did not differ in the second year (coinciding with the COVID‐19 pandemic) or 6 months after the program ended (West & Castro, 2023). Finally, in an analysis of Native Americans in the United States, the opening of a casino (bringing annual payments over multiple years to Native American families) was associated with reductions in symptoms of anxiety and heavy drinking (Wolfe, Jakubowski, Haveman, & Courey, 2012).

Studies of EITC payments are more consistent (than studies reviewed above) in demonstrating the benefits of increased EITC payments on psychological well‐being, including for workers without dependent children, some of whom were noncustodial parents (Courtin et al., 2022). In a study of 34,824 adults in the Panel Study of Income Dynamics, increased EITC refunds were associated with reductions in psychological distress, as measured by the Kessler‐6 scale (Kessler et al., 2002; Shields‐Zeeman, Collin, Batra, & Hamad, 2021). Using data spanning 1990–2017 collected as part of the Pregnancy Monitoring Risk Assessment Survey (*n* = 624,610) increases in the generosity of state‐level EITC policy relative to federal EITC policy were associated with decreases in binge drinking in 3 months prior to conception and in fewer reported postpartum depressive symptoms, particularly for single mothers with low levels of education (Morgan, Hill, Mooney, Rivara, & Rowhani‐Rahbar, 2022). A number of studies have shown that the subjective well‐being of mothers improved from before to after EITC expansions, including reductions in depression symptoms and increases in happiness and self‐esteem amongst women (*n* = 5,531) in the National Survey of Families and Households (Boyd‐Swan, Herbst, Ifcher, & Zarghamee, 2016) and reductions in the number of days (in the past month) that mothers participating in the Behavioural Risk Factors Surveillance Survey between 1993 and 2001 reported their mental health was poor (Evans & Garthwaite, 2014). The positive impacts of EITC expansions were restricted to married (versus unmarried) mothers, all of whom had no more than a high school education (Boyd‐Swan et al., 2016).

In summary, there is little evidence that one‐time, lump‐sum cash payments, particularly during the COVID‐19 pandemic, were associated with reductions in symptoms of depression and anxiety except for very low‐income households. It is unclear whether the null effects were driven by the size of payments, the frequency of payments (one‐time vs. recurring) or the fact that participants in the control group were also getting unprecedently large transfers of cash from the federal government in the form of stimulus checks and expanded tax credits over the study period. In contrast, there is more consistent evidence from studies of conditional cash payments, EITC studies and some unconditional cash transfer programs that cash transfers are associated with improvements in parent mental health. These studies, many of which were conducted prior to the pandemic, involve payments that families could count on to receive regularly (e.g., monthly or annually). Data are needed to test whether improvements in parent mental health associated with cash transfer programs lead to reductions in children's emotional and behavioural health problems (and vice versa).

#### Intimate partner relations

According to family strain theory, financial stress increases the risk of intimate partner violence because it contributes to feelings of powerlessness in men who respond by engaging in violence against women (Ahmadabadi, Najman, Williams, & Clavarino, 2020). There is substantial literature documenting that children who are exposed to intimate partner violence are at elevated risk for emotional and behavioural problems (Harold & Sellers, 2018; McTavish, MacGregor, Wathen, & MacMillan, 2016). A meta‐analysis of studies from low‐ and middle‐income countries found that cash transfer programs were associated with various forms of intimate partner violence, decreasing these by a statistically significant two to four percentage points (Baranov, Cameron, Contreras Suarez, & Thibout, 2021). In a review of studies (mostly from higher‐income countries) that used quasi‐experimental designs and randomized controlled trials to evaluate policies and interventions targeting socioeconomic conditions, four out of 11 studies that measured domestic violence reported significant reductions, with effect sizes that were small to moderate in magnitude (Courtin, Allchin, Ding, & Layte, 2019). In a review of studies that evaluated the impact of tax credits in the United States (e.g., CTC and EITC), these policies were also associated with reductions in intimate partner violence (Machado et al., 2024).

Thus, there is preliminary evidence that cash transfer programs will be associated with reductions in children's emotional and behavioural problems via decreases in children's exposure to intimate partner violence, although studies are needed to test this hypothesis. Moreover, studies in low‐ and middle‐income countries indicate the potential for cash transfer programs to backfire within the household if men perceive a threat to their financial control (Baranov et al., 2021).

#### Parent–child interactions

Family strain theory predicts that improvements in parents mental health and intimate partner relations will facilitate warm and affectionate interactions between parents and children and will reduce parents' use of harsh physical and nonphysical discipline, with benefits for children's emotional and behavioural health (Pinquart, 2017a, 2017b). In a systematic review of social safety net programs (including cash transfers) from low‐ and middle‐income countries, there was mixed evidence that these programs were associated with reductions in children's or adolescents' exposure to physical, emotional or sexual violence (Peterman, Neijhoft, Cook, & Palermo, 2017). In a review of conditional and unconditional cash transfer programs (Courtin et al., 2019), one study (in Gaza) measured harsh parenting, which was not associated with program receipt (Abu‐Hamad, Jones, & Pereznieto, 2014). To the best of our knowledge, studies have not measured parental warmth.

The evidence from high‐income countries that cash transfer programs improve parent–child interactions is also mixed. Across the five studies reviewed by Courtin et al. (2019) that measured adverse parenting practices (primarily harsh verbal and nonverbal discipline), only one reported a significant reduction associated with the intervention and across eight studies that looked at various measures of child maltreatment, only three detected reductions in any measure, with effect sizes that ranged from small to moderate (Courtin et al., 2019). In contrast, a review of studies that evaluated the impact of tax credits in the United States (e.g., CTC and EITC) showed that these policies were associated with reductions in child maltreatment rates at the family and state levels (Machado et al., 2024). Thus, it is not clear from these studies whether cash transfer programs are associated with less harsh and warmer and more affectionate parent–child relations, with stronger evidence for the impact of tax credits in the United States than for cash transfer programs in low‐ and middle‐income countries. There is, however, evidence from low‐ and middle‐income countries that cash transfer programs combined with behavioural change interventions targeting parenting do reduce forms of negative discipline, including corporal punishment and harsh verbal discipline (Betancourt et al., 2020; Premand & Barry, 2022).

## Conclusions and future directions

In high‐income countries like the US, cash transfers to lower‐income and some middle‐income families are implemented through a range of mechanisms, including tax credits, guaranteed income programs and industry‐related cash distributions. As shown in Table 1, the 10 studies we identified represented six unique samples. Three reported null findings, six reported positive effects (i.e., cash payments were associated with significant *reductions* in emotional and behavioural health problems) and one reported null findings for some outcomes and positive findings for others. Positive findings tended to be small to moderate in magnitude with, for example, children's problem behaviours reducing by 2%–5% of a standard deviation for every $1,000 increase in income (Hamad & Rehkopf, 2016) and physical fighting for boys reduced by 3.8% with every 10 percentage point increase in state EITC generosity (Dalve et al., 2022). These findings were often observed only amongst population subgroups (e.g., boys) in early childhood (Dalve et al., 2022; Rostad et al., 2020), although profits from the opening of a casino in the Qualla Boundary of Western North Carolina were associated with a 40% decline in adolescents' symptoms of behavioural problems (but little change in emotional problems; Costello et al., 2003). As the number of studies increases, it will be important for researchers to meta‐analyze results and to test whether factors like child age, child gender, child race/ethnicity, type of cash transfer, type of mental health outcome or size or frequency of the transfer account for heterogeneity in study findings.

Although the sample size is small, some conclusions can be drawn from our review of the literature. First, studies of low‐ and middle‐income countries have identified stronger impacts of regular cash payments (vs. annual or one‐time and lump‐sum payments) on a range of outcomes (Bastagli et al., 2016). Consistent with this finding, regular cash payments were more strongly associated with child and adolescent (and adult) mental health than one‐time, lump‐sum payments in the studies we reviewed. Regular payments are predictable, thus relieving stress and allowing families to plan for future expenses (Gennetian et al., 2023). For example, although findings from the CTC expansion were mixed, there was some evidence that the association between monthly CTC payments and reduced symptoms of anxiety increased in magnitude over time, potentially because their predictability afforded some stress relief or because payments accumulated over time (Kovski et al., 2023).

Second, consistent with findings from low‐ and middle‐income countries, the impact of cash payments on mental health and well‐being diminishes after program cessation (Wollburg et al., 2023). These findings suggest that cash transfer programs will be effective in reducing symptoms of mental health problems for children and adults only as long as they reduce material hardship. Such programs must either lift families out of poverty in an enduring way or remain a feature of the social safety net.

Third, effect sizes were small to moderate in magnitude and, in some cases, were observed only for the poorest families. For example, positive child behavioural outcomes were associated with only the 2009–2014 version of the CTC that included very low‐income households earning as little as $3,000 and introduced a lower refund threshold (Rostad et al., 2020). Lump‐sum payments of $1,000 may be as little as 9% of a family's income, although payments of this size may represent a substantially larger share of annual income for the poorest families (Pilkauskas et al., 2023). Thus, null findings and small effect sizes could be related to the relatively small size of cash transfers in the United States. Such findings would be consistent with studies from low‐ and middle‐income countries showing that larger payments (relative to baseline income) were more strongly associated with reductions in mental health problems and improvements in subjective well‐being (McGuire et al., 2022).

Another potential reason for small effect sizes is that psychopathology is not typically a target of poverty‐reduction programs. Instead, tax credits, industry‐related cash distributions and government stimulus programs are designed to achieve policy goals, from poverty alleviation and emergency response to work incentives. It is possible that programs designed with child health and well‐being as primary outcomes may produce larger effects. Innovative programs like Baby's First Years and Rx Kids (rxkids.org) target improvements in child outcomes and signal to parents the intended purpose of the cash (i.e., for children). This strategy is supported by research showing that labelling cash transfers for a particular good increases spending and consumption of the intended good (Beatty et al., 2014). Evaluations of these types of programs, especially when treatments are randomly assigned and a broad array of parent and youth sociobehavioural outcomes are collected, might provide the clearest demonstration yet of whether and how cash transfers affect child mental health.

Finally, our review highlights the challenges of evaluating the impact of cash transfer programs on child and adolescent mental health. Measures of young people's emotional and behavioural health are not systematically available in administrative datasets that could be linked with records of dividend payments or tax credits. Although there are nationally representative studies of young people's mental health, eligibility for tax credits is usually inferred in these studies (rather than measured directly) and information about other kinds of cash transfers is typically lacking. Dozens of guaranteed income studies were launched in the past several years (https://basicincome.stanford.edu/experiments‐map/), but many are small in size (typically involving 100–200 participants) and those that measure child and adolescent mental health may be underpowered to detect treatment effects. Meta‐analytic approaches may be required to draw conclusions about their impacts on children and families.

### Limitations

Our review of the literature is characterized by several limitations. First, many studies in our review included data from the same samples: three studies used data from the NLSY79CYA sample to test the impact of EITC policy changes and another three used data from the Great Smoky Mountain Study to test the impact of cash payments from the profits of a tribal casino. Thus, there is a need for studies based on a wider variety of samples to determine whether findings generalize across populations.

Second, although researchers typically use well‐validated assessments to measure symptoms of emotional and behavioural health problems such as the Center for Epidemiological Studies Depression scale (Radloff, 1977) or the Behaviour Problems Index (Peterson & Zill, 1986), the studies we reviewed did not measure mental health disorders per se. This limits the conclusions that researchers can draw about the impact of cash transfer programs on clinically significant levels of mental health problems. Even studies that combine poverty‐reduction and mental health interventions rely on well‐validated mental health screening measures or indices of antisocial behaviour (Blattman, Jamison, & Sheridan, 2017; Carpena et al., 2023), which may reflect the challenges of hiring and training interviewers to conduct time‐consuming and complex clinical assessments.

Third, the interpretation of the review's findings should be tempered by the lack of a systematic search strategy to identify cash transfer programs implemented in the United States. Given the number of new cash transfer programs that have been implemented and evaluated in the last few years, some relevant studies may have been missed. The review's focus on only US studies may also miss important studies from other high‐income countries, though studies from Canada and the United Kingdom are cited (Beatty et al., 2014; Milligan & Stabile, 2011). We argue that features of the social safety net in the United States are sufficiently unique to merit a narrow focus on the impact of cash transfers in this context and we provide new information on the effects of US cash transfer programs on child and adolescent mental health, including potential mechanisms of effect.

Fourth, many of the studies we reviewed were conducted during the COVID‐19 pandemic. Null findings from these studies are particularly difficult to interpret, given the unprecedented shock to the whole economy, with most services and systems forced to shift what they provided and how they provided it and the federal government offering unprecedented levels of support to families in the form of stimulus payments and temporary EITC and CTC expansions.

### Directions for future research

Reviews of cash transfer programs in low‐ and middle‐income countries have concluded that larger unconditional cash transfers (relative to baseline income) are more impactful than smaller ones. In low‐ and middle‐income countries, cash transfer programs boost income by as much as 200% (West et al., 2023). Cash payments of this magnitude are unrealistic in the United States, where incomes are substantially higher. With a large (and growing) number of guaranteed income studies in the pipeline that range in the amount and duration of payments to families, there are important opportunities for researchers to learn more about the amount of money required (in absolute or relative terms) to promote the most positive mental health outcomes.

Many cash transfer programs are designed with a theory of behaviour change, which should allow researchers to test potential mechanisms by which cash transfers benefit children and families. As reviewed above, these mechanisms are likely to involve increases in parental investments in children (both in terms of expenditures and time) and improvements in parental mental health, parent–child interactions and relationships with intimate partners. Formal tests of mediation will be required to test these hypothesized mechanisms, including complex models that accommodate bidirectional effects (e.g., improvements in child outcomes leading to better parent mental health) and models that test cascading effects (e.g., improvements in caregiver mental health leading to more positive parent–child interactions and, in turn, better child emotional and behavioural health). Support for such associations was evident in some interviews with Family Rewards participants who noted that the program incentivized their children to attend school more regularly and to earn better grades – factors the parents associated with increases in their own psychological well‐being (Miller et al., 2016).

Future research can also make use of administrative data to better understand the impacts of cash transfer programs on children's exposure to violence, by measuring children's exposure to violent and nonviolent crime in their neighborhoods and their risk of contact with child protective services. Studies exploiting natural experiments or even randomly assigning treatment are likely to provide the highest quality causal evidence on both mechanisms and outcomes, especially when cash transfer receipt, amount and timing can be confirmed rather than estimated.

Current studies of cash transfers and child and adolescent mental health often involve samples that range widely in age. This is particularly true of studies of the EITC and CTC. The literature on poverty and children's outcomes has shown that exposure early in life to poverty is more strongly associated with cognitive and behavioural outcomes across the life course than exposure in adolescence (Duncan, Brooks‐Gunn, Yeung, & Smith, 1998; McFarland, 2017). There is an opportunity to test whether cash transfer programs have stronger impacts at certain points in childhood or adolescence than others and whether these timing effects depend on the outcome in question. A limitation of these efforts will be the difficulty in disentangling timing from chronicity. Given that cash transfer programs are targeted at poor and working‐class families, older children are likely to have been exposed to poverty for longer than younger children by the time their families receive cash payments. A challenge in this research will be detecting effect modifiers when these represent combinations of variables (e.g., girls vs. boys at particular points in development or in particular arms of a trial).

Unconditional cash transfer programs in the United States are taking new forms in addition to guaranteed income pilots. Recently, the Office of Policy Development and Research at the Department of Housing and Urban Development called for pilots that compare direct‐to‐tenant payments with the Housing Choice Voucher program, which is the US federal government's largest form of housing assistance to low‐income renters (McCabe & Shroyer, 2023). The PHLHousing+ program is the first example of this kind of pilot; it will compare children and adults in households receiving monthly cash payments or housing vouchers to children and adults in households wait‐listed for public housing assistance (Reina, Fowle, Mulbry, Fortenberry, & Jaffee, 2024). In addition to potential mediators already identified in other studies, direct comparisons of cash‐based versus in‐kind forms of support may shed light on new mechanisms by which programs have positive effects (e.g., by promoting autonomy in how households deal with housing or financial precarity; Gennetian et al., 2021). Other programs combine cash transfers with parenting or mental health interventions. Such studies can identify individual and combined effects of program components (Blattman et al., 2017; Carpena et al., 2023), with sustained effects (Blattman, Chaskel, Jamison, & Sheridan, 2023), although some data from low‐ and middle‐income countries fail to show the added value of mental health interventions on adult mental health; Haushofer, Mudida, & Shapiro, 2020). The ALIVE study will provide new data on how antipoverty programs plus psychosocial interventions affect adolescent mental health (Lund et al., 2023). Existing and planned studies have all been conducted in low‐ and middle‐income countries (Liberia, Nepal, Colombia and South Africa); it is unclear if the effects would replicate in higher‐income countries.

### Implications for practitioners

Clinicians are increasingly aware of the need to address social determinants of health (like economic well‐being; e.g., Allen, Balfour, Bell, & Marmot, 2014). This awareness includes a recognition that patients' symptoms may originate in the patient's socioeconomic circumstances and in the interaction between such circumstances and a person's innate or early emerging cognitive and temperamental traits. Social workers are common in primary healthcare settings, helping to connect patients and their families with needed resources (Rowe, Rizzo, Vail, Kang, & Golden, 2017). Some hospitals have established medical‐legal partnerships to provide legal support to clinicians and social workers to address structural problems that negatively impact patients' health (e.g., risk of eviction, loss of benefits, etc.; Tobin‐Tyler & Teitelbaum, 2019). In addition, some hospital systems have developed medical–financial partnerships that are designed to connect patients to community partners offering wealth‐building services (Dalembert, Fiks, O'Neill, Rosin, & Jenssen, 2021). These interprofessional teams have the potential to improve patient care, particularly if there are reimbursement streams to support their work (Rowe et al., 2017).

### Conclusion

Although cash transfer programs are common poverty alleviation tools in low‐ and middle‐income countries, their popularity in higher‐income countries like the United States is more recent. With a growing number of cash transfer programs in the pipeline that vary in length, payment structure and target population, there are rich opportunities to test the conditions under which they are most strongly associated with child and adolescent mental health. Efforts to draw strong policy recommendations may require meta‐analyses of results, and the best‐designed studies will enable tests of hypothesized mediators of program effects.

**Key points**

- Although cash transfer programs are gaining in popularity in the United States, little is known about their effects on child and adolescent mental health in higher‐income countries or the mechanisms by which they might improve child and adolescent mental health.
- The evidence from quasi‐experimental and experimental studies of cash transfer programs and child and adolescent mental health in the United States is based on a small set of samples, with the majority of studies showing benefits to mental health.
- Efforts to draw strong policy recommendations may require meta‐analyses of results, and the best‐designed studies will enable tests of hypothesized mediators of program effects.

## Data Availability

Data sharing is not applicable to this article as no new data were created or analyzed in this study.
